# The High Level of RANKL Improves IκB/p65/Cyclin D1 Expression and Decreases p-Stat5 Expression in Firm Udder of Dairy Goats

**DOI:** 10.3390/ijms24108841

**Published:** 2023-05-16

**Authors:** Zhen Gao, Dan Shao, Chunrui Zhao, Haokun Liu, Xiaoe Zhao, Qiang Wei, Baohua Ma

**Affiliations:** 1 Key Laboratory of Animal Biotechnology of the Ministry of Agriculture, Northwest A&F University, Yangling, Xianyang 712100, China; 2 College of Veterinary Medicine, Northwest A&F University, Yangling, Xianyang 712100, China

**Keywords:** Guanzhong dairy goats, firm udder, RANKL, milk protein synthesis

## Abstract

Udder traits, influencing udder health and function, are positively correlated with lactation performance. Among them, breast texture influences heritability and impacts on the milk yield of cattle; however, there is a lack of systematic research on its underlying mechanism in dairy goats in particular. Here, we showed the structure of firm udders with developed connective tissue and smaller acini per lobule during lactation and confirmed that there were lower serum levels of estradiol (E_2_) and progesterone (PROG), and higher mammary expression of estrogen nuclear receptor (ER) α and progesterone receptor (PR), in dairy goats with firm udders. The results of transcriptome sequencing of the mammary gland revealed that the downstream pathway of PR, the receptor activator of nuclear factor-kappa B (NF-κB) ligand (RANKL) signal, participated in the formation of firm mammary glands. During the culture of goat mammary epithelial cells (GMECs), high RANKL level additions promote the Inhibitor kappaB (IκB)/p65/Cyclin D1 expression related to cell proliferation and decrease the phosphorylated signal transduction and transcription activator 5 (Stat5) expression related to milk-protein synthesis of GMECs, which is consistent with electron microscope results showing that there are fewer lactoprotein particles in the acinar cavity of a firm mammary. Furthermore, co-culturing with adipocyte-like cells for 7 d is beneficial for the acinar structure formation of GMECs, while there is a slightly negative effect of high RANKL level on it. In conclusion, the results of this study revealed the structure of firm udders structure and confirmed the serum hormone levels and their receptor expression in the mammary glands of dairy goats with firm udders. The underlying mechanism leading to firm udders and a decrease in milk yield were explored preliminarily, which provided an important foundation for the prevention and amelioration of firm udders and improving udder health and milk yield.

## 1. Introduction

Udder traits, an important factor influencing udder health and function, have been demonstrated a positive correlation with lactation performance [1,2]. Among them, breast texture is discussed without a quantitative and specific definition of the firmness of a healthy udder. Based on the reports of Rosenberger and Houe et al., udders are classified as soft, firm, and hard without atrophy or mastitis, according to tissue condition [3,4], which is positively correlated with milk yield during 305 d lactation in Holstein cattle [5]. It is important to choose appropriate udder traits for ensuring udder health and improving milk performance, and the research on breast texture mainly focuses on its heritability and the impacts on the milk yield of cattle [6,7,8]. In addition, udder morphology and firmness are considered to be related to mastitis susceptibility [9,10,11,12,13]. However, there is a lack of in-depth research on the underlying mechanism of firm udders and the effects on udder structure and lactation, particular in dairy goats, which further emphasizes the importance of interpreting its formation and influence to prevent and deal with firm udders. To probe the udder structure of firm udders, paraffin sections and ultrathin sections were performed in this research.

Epithelial ducts in mammary glands are surrounded by adipose tissue. Mammary epithelium undergoes circulatory remodeling during pregnancy, lactation, and degeneration, a process that is accompanied by adipose remodeling [14,15,16]. During pregnancy, the mammary epithelium undergoes a massive expansion characterized by ductal side-branching and alveoli formation: the milk-secreting units. Epithelial intrinsic progesterone receptor (PR) signaling is required for side branching and alveologenesis, and adipose tissue strongly promotes the growth of mammary epithelial cells (MECs) by providing structural support and regulatory signals [14,17,18]. PRs, which are composed of two proteins expressed from a single gene as a result of transcription from two alternative promoters, are expressed in both epithelial and stromal compartments in mouse mammary glands [19]. There are two distinct mechanisms underlying progesterone (PROG)-induced proliferation in the mammary gland: (i) a small wave encompassing PR^+^ cells and requiring cyclin D1; and (ii) a large wave comprising mostly PR^−^ cells and relying on the tumor necrosis factor (TNF) family member, receptor activator of nuclear factor-kappa B (NF-κB) ligand (RANKL) [20]. RANKL binds to its receptor, receptor activator of nuclear factor κB (Rank) expressed on the surface of MECs, activating inhibitor κB kinase (IKK)-α and leading to Inhibitor kappaB (IκB) α proteasome degradation and separating from NF-κB. Then, p65 subunit migrates to the nucleus and induces cyclin D1 transcription and the proliferation of MECs [21,22,23]. Taken together, we speculated that there was an abnormal RANKL signal pathway in firm udders that participated in its formation, regulated by PROG and PR. The ELISA test and immunohistochemistry (IHC) staining were performed to determine the levels of hormones and their receptors, and transcriptome sequencing was executed to examine this speculation. To explore the underlying mechanism of firm udders from a cell level, further signal pathways regulating GMECs proliferation and function were also investigated.

There is a significant difference between adipose tissue of the breast and other parts. Mature adipocytes in mammary glands exhibit unusual cellular plasticity, transforming into secretory MECs during the “adipo-epithelial trans-differentiation” process [24,25,26]. Adipose tissue is now known to have a multitude of endocrine and developmental functions [27,28]. The effects of mammary adipose on MECs are through hormone and paracrine factors, including prolactin (PRL), estradiol (E_2_), insulin-like growth factor-I, leptin and adiponectin [14,29,30]. The exact molecular mechanisms of the interaction between mammary adipocytes and MECs are unclear. PRL, which plays an important role in alveologenesis and differentiation of MECs into lactating cells during late pregnancy, is expressed and released by mammary adipose [31,32,33]. In lactating mice, signal transduction and transcription activator 5 (STAT5), a transcription factor family in cytoplasm, regulates MECs development and milk-protein synthesis [34,35]. PRL binds to the prolactin receptor (PRLR) to induce JAK2 activation, then activate downstream STAT5 and transfer to the nucleus to regulate target genes transcription, such as epidermal regulator (Ereg) and whey acid proteins (WAP) [36,37]. The aim of this study was to explore whether a high level of RANKL was the key mechanism contributing to firm udder formation in dairy goats, the decrease of milk yield, and the effects of adipocytes on this biological process in mammary glands.

## 2. Results

### 2.1. The Firm Udder Structure of Guanzhong Dairy Goats

**Figure 1:**
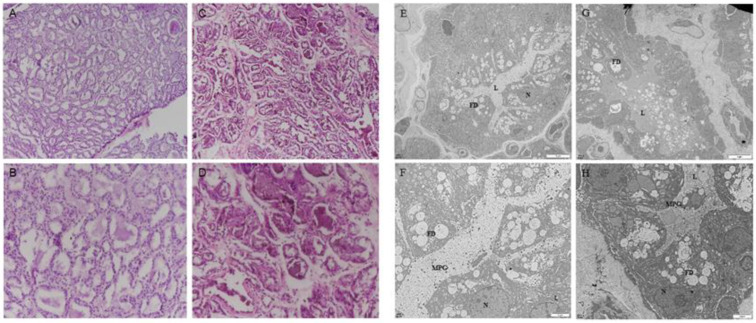
The firm udder structure of Guanzhong dairy goats: (**A**–**D**) HE staining of normal (**A**,**B**) and firm (**C**,**D**) mammary gland sections of Guanzhong dairy goats (**A**,**C**: 100×, **B**,**D**: 200×); (**E**–**H**) transmission electron micrograph of normal (**E**,**F**) and firm (**G**,**H**) mammary gland sections of Guanzhong dairy goats; N: Nuclear; L: Acinar cavity; FD: lipid droplets; MPG: milk protein particles; Scale bars: 5 μm (**E**,**G**); Scale bars: 2 μm (**F**,**H**).

To define the firm udder structure of Guanzhong dairy goats, paraffin embedment and HE staining were performed. Compared with the normal mammary gland structure of Guanzhong dairy goats, during lactation, there were more mammary lobules in the firm udder, and in each mammary lobule there were less acini with more connective tissue between acini, as shown in Figure 1A–D. 

The transmission electron microscope results showed that the acinar cavity and GMECs in the normal mammary gland were filled with a large number of lipid droplets and milk protein particles, and the lactating GMECs arranged regularly and closely (Figure 1E,F). Conversely, in firm udders, there were fewer milk-protein particles in the cinar cavity and lactating GMECs were arranged regularly but not tightly (Figure 1G,H). In addition, in the normal mammary gland, the uniform arrangement of collagen fibers in the connective tissue between mammary acinar cavities was vertical and horizontal, and reticular fibers were crosslinked between collagen fibers, while in the mammary gland with high firmness, the reticular fibers, with proliferation were filled with irregular collagen fibers. 

### 2.2. Hormones Serum Levels and Receptor Mammary Expression in Goats with Firm Udder

To determine whether or not the unusual mammary structure was related to E_2_, PROG and PRL, hormones levels in serum and their receptor expression in mammary gland of Guanzhong dairy goats with firm udder were performed further by IHC staining and ELISA. 

**Figure 2:**
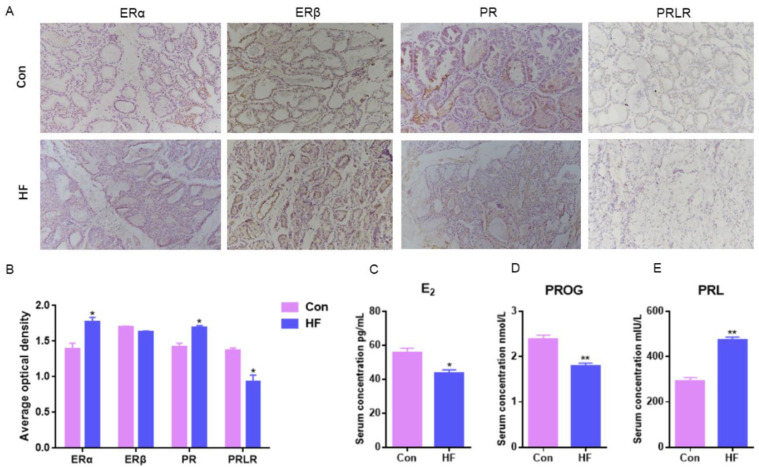
Hormone serum levels and receptor expression in mammary gland of dairy goats with firm udders: (**A**,**B**) the expression levels of ERα, ERβ, PR and PRLR in normal and firm mammary gland sections, respectively (200×); (**C**–**E**) the serum levels of E_2_, PROG and PRL in goats with normal and firm mammary glands, respectively. Con: control; HF: firm udder. The asterisk indicates significant difference (*p* < 0.05). The double asterisk indicates highly significant (*p* < 0.01).

E_2_ could combine with ERα without ERβ to regulate mammary duct growth and morphogenesis, compared with normal goats, ERα mammary expression in the firm udder was significantly higher (*p* < 0.05) and there was no significant difference in ERβ between the normal and firm udder (Figure 2A,B). PR mammary expression in the firm udder was significantly higher than in the normal mammary gland (*p* < 0.05) (Figure 2A,B), which contributed to the proliferation and interstitial hyperplasia in the mammary gland with high firmness. Compared with normal goats, PRLR mammary expression in the firm udder was lower than in the normal udder, which was unbeneficial to alveoli development (Figure 2A,B). Contrary to the above results, the serum E_2_ and PROG levels of goats with firm udders were both significantly lower than those of normal goats, and the PRL level was significantly higher than that of normal goats (Figure 2C–E). It may be because at a lower level of E_2_ and PROG, more ERα and PR expressed in mammary glands with high firmness were needed to maintain normal function.

### 2.3. Transcriptome Sequencing and Validation of Mammary Gland in Guanzhong Dairy Goats with Firm Udder

**Figure 3:**
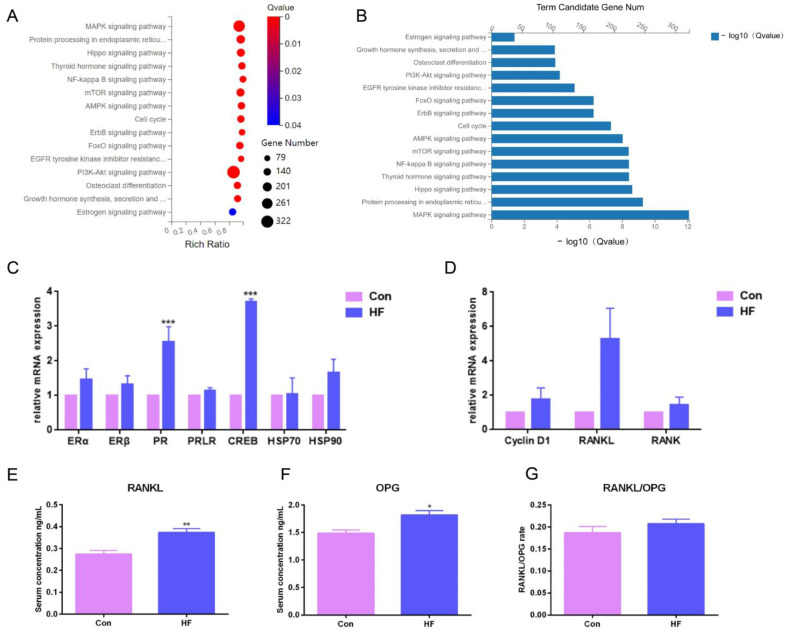
Pathway-enrichment analysis of differentially expressed genes in the mammary glands of dairy goats with firm udders: (**A**,**B**) the results of KEGG pathway enrichment in firm mammary gland; (**C**,**D**) the mRNA expression of downstream genes of estrogen and progesterone in normal and firm mammary glands; (**E**,**F**) the serum levels of RANKL and OPG in goats with normal and firm mammary glands, respectively; (**G**) the ratio of RANKL and OPG levels. The asterisk indicates significant difference (*p* < 0.05). The double asterisk indicates highly significant (*p* < 0.01). Three asterisks indicate extremely significant (*p* < 0.001).

To investigate the underlying signaling pathway contributing to firm udder formation, the transcriptome sequencing of mammary glands from three normal Guanzhong dairy goats and three Guanzhong dairy goats with firm udders was performed, and the data were further analyzed through KEGG pathway-enrichment analysis (Figure 3A,B). Through mRNA differential expression analysis, the gene networks related to cell proliferation, material metabolism and immunity were found. There was a large-scale change in the mRNA expression of mammalian target of rapamycin (mTOR) signal pathway related to milk protein synthesis, lipid metabolism, autophagy, cell survival and proliferation, and mitogen-activated protein kinases (MAPK), Phosphatidylinositol 3-kinase (PI3K)—protein kinase B (AKT), adenosine 5′-monophosphate (AMP)-activated protein kinase (AMPK) and human epidermal growth factor receptor (ErbB) signal pathways directly related to the mTOR signal pathway. In addition, it was found that the mRNA levels of downstream genes of E_2_ binding to its receptor upregulated, such as PR, matrix metalloproteinase 9 (MMP 9), keratin 19 (Krt19), retinoic acid receptor (RAR α) and cAMP response element binding protein (CREB), and mRNA levels of RANKL/Cyclin D1 that was downstream of PR and related to the MECs proliferation upregulated also. These differences, indicating that the formation of mammary glands with high firmness was closely related to the signal pathway regulated by E_2_ and PROG, and PR/RANKL/Cyclin D1 signaling pathway, upregulated by higher level of PR expression and PRL stimulation, were focused on in this study. 

To validate the RNA-seq data, the expression of ER and PR responsive genes was measured by qRT-PCR. Consistent with the RNA-seq data, qRT-PCR results showed that mRNA expression levels of PR downstream signal, such as RANKL, were upregulated (Figure 3C,D). Then, the serum levels of RANKL and its decoy soluble receptor OPG in dairy goats with firm udders were tested by ELISA. The RANKL and OPG levels of the goats with firm udders were both significantly higher than those of normal goats (*p* < 0.05), and the RANKL/OPG level was higher than that of normal goats (Figure 3E–G), which both suggested that in dairy goats, firm udder formation may be closely related to the RANKL/p65/cyclin D1 signal pathway.

### 2.4. The Effects of High RANKL Level on Primary GMECs Proliferation, Function and Formatting Acini 

**Figure 4:**
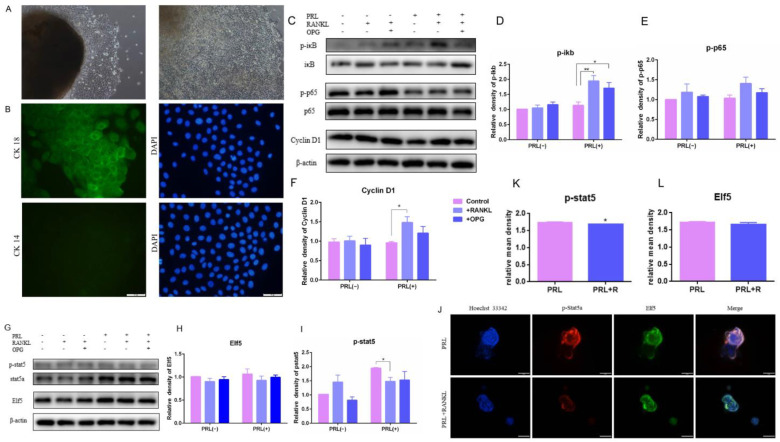
The protein expression of IκB/p65/Cyclin D1 and p-Stat5 and the test of acini structure of GMECs with high RANKL level: (**A**) the primary GMECs migrated out from the mammary gland after 8 d and 12 d of culture, respectively (100×); (**B**) the appraisal of GMECs by immunofluorescence staining of CK18 and CK14; (**C**–**F**) the protein-expression levels of IκB/p65/Cyclin D1 pathway; (**G**–**I**) the protein-expression levels of Elf5 and p-stat5; (**J**) the immunofluorescence staining of acini structure with p-stat5 (**K**) and Elf5 (**L**). Scale bars: 50 μm. The asterisk indicates significant difference (*p* < 0.05). The double asterisk indicates highly significant (*p* < 0.01).

The upregulated RANKL/p65/cyclin D1 signal pathway in the firm udders was identified based on the results of the transcriptome sequencing, and the effects of high RANKL on GMECs proliferation, function and formatting acini were explored to elucidate the contribution of upregulated RANKL in firm udders. GMECs were isolated using the tissue block culture method. After 5–7 d of culture, polygonal and tightly arranged epithelioid cells began to creep out around the tissue block, and no fibroblasts were seen migrating from the tissue block (Figure 4A left); After 1 of culture, multiple cells in the dish formed a multi-layer structure, and cells were subcultured (Figure 4A right); GMECs grew to a cobblestone-like shape after confluence. Cytokeratin 18 (CK18) is a specific marker for MECs, while cytokeratin 14 (CK14) is a specific marker for mammary myoepithelial cells. Based on this characteristic, we identified GMECs according to immunofluorescence staining, and the results showed that GMECs isolated by the tissue block culture method expressed CK18, but did not express CK14 (Figure 4B).

Activation of IKK-α results in IκB α proteasome degradation and NF-κB isolation, migration to the nucleus, and cyclin D1 transcription. The expression of phosphorylated IκBα, phosphorylated p65 and cyclin D1 in GMECs was upregulated with the high dose of RANKL treatment for 24 h, indicating that RANKL triggered IκB/p65/Cyclin D1 and promoted GMECs proliferation, and that OPG treatment could reverse the effect of RANKL (Figure 4C–F). However, the expression of phosphorylated stat5 in GMECs and acini structure-forming by GMECs was downregulated significantly with lactation induction (*p* < 0.05), indicating that high RANKL levels inhibited milk-protein synthesis in GMECs (Figure 4G,I–K). Then, the formation of acini structure was detected and results displayed that the Elf5 protein expression of GMECs decreased with insignificant difference (*p* > 0.05) and the structure of GMECs-formatting in Matrigel was more irregular under high RANKL levels, which suggested that the formation of the acini structure was affected slightly after 24 h treatment with the high RANKL level (Figure 4G,H,J,L). 

### 2.5. The Effects of GMECs and Adipocyte-like Cells Co-Culturing on IκB/p65/Cyclin D1 and Elf5 in GMECs

**Figure 5:**
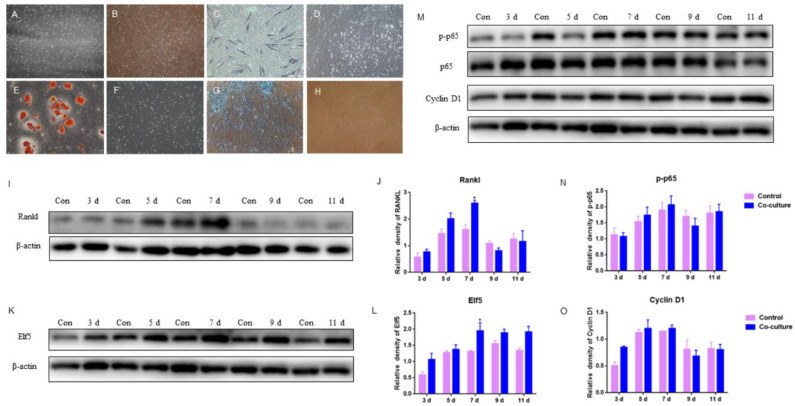
Effects of GMECs and adipocyte-like cells co-culturing on IκB/p65/Cyclin D1 pathway and Elf5 in GMECs: (**A**,**B**) the primary and 10th generation of ADSCs isolated from mammary glands, respectively (100×); (**C**,**D**) ADSCs were stained with alkaline phosphatase (ALP) after osteogenic induction and basic culture, respectively (100×); (**E**,**F**) ADSCs were stained with oil red O staining after lipogenic induction and basic culture, respectively (100×); (**G**,**H**) ADSCs were stained with Alcian blue after chondrogenic induction and basic culture, respectively (100×); (**I**,**J**) the protein level of RANKL expressed by GMECs after co-cultured with differentiated adipocyte-like cells after adipogenic induction; (**M**–**O**) the protein level of p-p65 and Cyclin D1 expressed by GMECs after co-cultured with differentiated adipocyte-like cells after adipogenic induction; (**K**,**L**) the protein expression of Elf5 expressed by GMECs after co-cultured with differentiated adipocyte-like cells after adipogenic induction. The asterisk indicates significant difference (*p* < 0.05).

According to the firm udder structure of developed connective tissue and smaller acini per lobule, GMECs were co-cultured with adipocyte-like cells induced by ADSCs from the mammary gland to examine the effects of mammary adipose on acini formation. The mammary adipose of dairy goats was digested by type I collagenase to isolate and culture ADSCs. On 6 d of culture, the cells were spindle-shaped with mitosis and spread all over the culture dish with clear boundaries (Figure 5A). After being passed to the 10th generation, cells still maintained fibroblast-like cell morphology with strong proliferative activity (Figure 5B).

After osteogenic induction for 14 d, ADSCs were stained with alkaline phosphatase (ALP), a marker of osteogenic differentiation. It was observed that some cells were bluish-purple (Figure 5C), indicating that ADSCs exhibited osteogenic phenotype. Adipocyte-like cells were obtained after 14 d of lipogenic induction, and obvious red lipid droplets could be observed with oil red O staining (Figure 5E); After chondrogenic induction for 14 d, cells were stained blue by Alcian blue (Figure 5G), indicating that ADSCs exhibited chondrogenic phenotype. The above results indicated that the ADSCs isolated from goat mammary glands were adipose stem cells with typical characteristics of successful adipogenic differentiation, osteogenic differentiation, and chondrogenic differentiation.

After the adipogenic induction of ADSCs isolated from goat mammary gland for 14 d, the differentiated adipocyte-like cells and GMECs were co-cultured with transwell^TM^ for 3, 5, 7, 9, and 11 d, respectively. The results showed that the protein level of RANKL expressed by GMECs was increasing by 7 d and it was higher in the co-culture group than in those of the control, especially on 7 d (*p* < 0.05); after 7 d, it was lower in the co-culture group than in those of the control (*p* > 0.05), which indicated that adipocyte-like cells improved the RANKL expression of GMECs in 7 d (Figure 5I,J). In addition, there was a light impact on GMECs proliferation because there were slightly higher and lower protein levels of p-p65 and Cyclin D1 expressed by GMECs in the co-culture group by 7 d and after 7 d, respectively (*p* > 0.05) (Figure 5M–O). On the other hand, the protein expression of Elf5 in GMECs co-cultured on different days was always higher than that in the control, especially on 7 d (*p* < 0.05) (Figure 5K,L), indicating that co-culturing with adipocyte-like cells to 11 d has been beneficial for the acinar structure formation of GMECs with lactating function.

## 3. Discussion

Improving milk yield and milk quality, and solving the problems affecting the lactation of dairy goats, are among the main pursuits of researchers. Breast texture can reflect on the udder’s internal structure and the development of dairy goat mammary glands, and also influence lactation, to a certain extent. Udder firmness is characterized by stronger connective tissue in which udder parenchyma with alveoli aggregations is partitioned into lobules by connective tissue septa [38]. In this research, the results of HE staining showed that the internal acini in firm udder were underdeveloped and the acinar cavity was small, so there was less lactoprotein in acinar cavity, which was also confirmed by the electron microscope results. In addition, the connective tissue between acini in the firm udder was developed, which may jointly lead to the firm texture of a breast. ELISA results showed that the serum level of E_2_ and PROG was significantly lower than in normal dairy goats (*p* < 0.05), and IHC results showed that there were more ERα and PR in mammary glands with high firmness in order to maintain tissue normal function during lactation under the lower level of E_2_ and PROG. The serum level of PRL in dairy goats determines the start of lactation but is not the key to maintaining lactation. Therefore, even when the serum level of PRL varied greatly, there was no significant difference in PRLR level in mammary glands.

The subsequent transcriptome sequencing results showed that in addition to mTOR, MAPK, PI3K-AKT, AMPK and ErbB signaling pathways, the downstream signal pathways of ER and PR also upregulated to different degrees, which also corresponded to the IHC results. Among them, the significant upregulation of PR/RANKL/Cyclin D1 has attracted our attention. Consistent with the IHC and ELISA results of high PRL serum levels, it has been reported that PRL stimulation could improve RANKL expression, which may contribute to firm udder formation together with high PR expression in mammary glands [39]. The binding of RANKL and RANK triggers recruitment of TNF receptor- associated factor adaptor proteins and activates downstream signaling pathways (such as NF-κB, PI3K-AKT, and the MAPK cascade) [40]. The research on bone tissue suggests that estrogen withdrawal could promote the maturation and differentiation of osteoclasts by increasing the RANKL/OPG ratio and thus inducing osteoporosis [41]. In addition, RANKL is a key regulatory factor for MECs proliferation and differentiation and drives mammary morphogenesis during pregnancy and lactation. RANKL has been demonstrated to be a direct PR target gene with multiple functional P4 responsive enhancers that bind PR in a hormone-dependent manner [42,43,44,45]. It has been illustrated that during mammary lactational morphogenesis responsible for progesterone, RANKL elicits proliferation by a cell-intrinsic and a paracrine mechanism [20]. Corresponding to the transcriptome sequencing results, the serum RANKL level of dairy goats with firm udders was detected by ELISA, which indicated that the RANKL level in these dairy goats’ serum was significantly higher than that of normal goats. In order to explore the effects of high-level RANKL in mammary glands with high firmness, we continue to carry out follow-up research. RANKL, with its cellular receptor RANK, and the decoy soluble receptor OPG, which can neutralize the effects of RANKL by binding to RANKL and prevent it from activating RANK, have been characterized for their functions in mammary glands [46]. The distribution and expression of RANKL protein is strictly regulated by hormone levels, such as PROG, E_2_, and PRL [47]. PROG can directly stimulate the proliferation of PR-positive MECs by increasing Cyclin D1 activity, and also promotes the proliferation of PR-negative MECs through RANKL released by PR-positive cells [20]. Similarly, after being treated with high doses of RANKL for 24 h, the upregulation of p-IκB and Cyclin D1 expressed by MECs indicates that RANKL plays a role in promoting MECs proliferation, and that OPG treatment can reverse the upregulation of p-IκB and Cyclin D1 controlled by RANKL.

In order to explore further whether high RANKL levels affect the structure and milk synthesis of mammary glands, we conducted research on GMECs treated with high doses of RANKL. In mammary glands, much of the PROG action is mediated by RANKL, including ductal side branching and Elf5 expression favoring the differentiation of progenitor cells towards secretory lineage [48]. Ectopic expression of RANKL in mammary epithelium is demonstrated to elicit ductal side branching and alveologenesis, and the overexpression of its cognate receptor RANK resulted in increased proliferation [49,50]. Consistent with the above results, in this research, a similar alveolar structure was exhibited in normal and firm mammary glands, and lactation ability was examined with high RANKL levels. Combined with the electron microscope results, there are fewer lactoprotein particles in the mammary acinar cavity with a firm texture. To explore the reason why lactoprotein synthesized by GMECs in this type of udder decreased, we further tested the p-stat5 expression and the ability form the acinar structure of GMECs exposed to high RANKL levels. In this research, the p-stat5 expression of the GMECs exposed to high RANKL levels was significantly reduced, which indicated that the ability of lactoprotein synthesis decreased significantly under high RANKL levels, and could lead to low milk yield or even to the low milk quality of dairy goats with firm udder textures. Consistently, in cow MECs, RANKL overexpression markedly decreased the STAT5a phosphorylation level [39]. However, the Elf5 expression and acinus-like structure formation under 3D culture conditions are affected without a significant difference, which suggests that the ability of GMECs to form an acinar structure is affected slightly, and this may be the reason for the similar appearance of normal and firm udders. Lactogenic hormones, such as PRL, bind to their receptors and regulate lactation and milk proteins through the JAK-STAT pathway [33,36,37,51], among which *STAT5A* has been found to be the crux of regulating milk yield, milk fat and protein [38,40]. Moreover, during pregnancy, Elf5-knockout mammary epithelium completely failed to initiate alveologenesis and lactogenic differentiation with a decreased level of phosphorylated Stat5 [52,53]. Elf5, a GTPase activating protein, is a key PRL-regulated gene that mediate alveolargenesis and PRL-driven mammary development. Re-expression of Elf5 in PRLR nullizygous mammary epithelium restores lobuloalveolar development and milk production, demonstrating that Elf5 is a transcription factor capable of substituting for prolactin signaling [54]. Despite RANKL/NF-κB/ Cyclin D1 axis playing an important role in MECs proliferation and alveolargenesis, it has been reported that enhanced RANKL/Rank signaling affects lactogenic differentiation in MECs by inhibiting the PRL-induced activation of Stat5 and expression of Elf5 required for lactation, and that RANKL inhibition at mid-pregnancy resulted in increased Elf5 expression and activation of Stat5, leading to a premature secretory differentiation [55,56]. Similar to the above reports, although the signaling pathway of RNAKL regulated MECs proliferation, our results with the RANKL treatment also decreased Stat5 activation, which may be the reason why milk production was affected, and also explained the decrease in the milk yield of dairy goats with firm udders.

According to the HE staining results, the connective tissue between acini is more developed in the firm udders of dairy goats. In order to explore the effects of developed connective tissue between acini on the structure and function of acini in firm udders, we conducted the co-culture of GMECs, and adipocyte-like cells induced by ADSCs derived from breasts. Based on the discovery of high RNAKL levels in firm udders, we also tested the RANKL expression of GMECs after co-culturing with adipocyte-like cells induced by ADSCs derived from goat mammary. Although the co-culture could increase the expression level of RANKL in GMECs, whether or not RANKL in firm mammary glands is derived from adipocytes apart from GMECs is still unclear. In addition, whether or not the cause of the RANKL increase is adipocytes promoting RANKL expression in GMECs, or the E_2_ level affecting RANKL level, or both, requires further research. In this research, there was a light impact on GMECs proliferation because there were slightly higher and lower protein level of p-p65 and Cyclin D1 expressed by GMECs in the co-culture group by 7 d, and after 7 d, respectively (*p* > 0.05). Consistent with this result, Marzan et al. reported that there are factors secreted by adipocytes promoting proliferation and migration through primary adipocytes or differentiated adipocyte lines co-culturing with MECs in vitro [57]. Furthermore, it has been reported that direct epithelial–adipocyte interactions are required for mammary gland development, but not for milk production or fertility [30]. A study on rats also found that mammary adipocytes induced acinar morphogenesis and enhanced the differentiation and casein and lipids accumulation of MECs mediated by diffusible paracrine factors [58,59]. Similarly, this research also showed that Elf5 expression of GMECs co-cultured on different days was always higher than that in the control, especially on 7 d (*p* < 0.05), indicating that co-culturing with adipocyte-like cells to 11 d has been beneficial for the acinar structure formation of GMECs with lactating function. Therefore, the effects of adipose on GMECs function is beneficial in this microenvironment and it is worthy of further research with regard to its role in the formation and function of firm udders.

## 4. Materials and Methods

### 4.1. Chemicals and Guanzhong Dairy Goat

All chemicals for this study were purchased from Sigma-Aldrich (St. Louis, MO, USA) unless stated otherwise. Plastic dishes and tubes were obtained from Nunc (Roskilde, Denmark). All mammary-gland samples in this research were obtained from three normal mammary glands and three firm mammary glands of Guanzhong dairy goats, which were given general anesthesia and treated with surgery provided by Hongxing MEILING Dairy Co., Ltd. (Weinan, China) in Fuping county, Shaanxi province, China. All blood samples for the ELISA test were obtained from 20 normal Guanzhong dairy goats and 20 Guanzhong dairy goats with firm udders, provided by Hongxing MEILING Dairy Co., Ltd. also. These Guanzhong dairy goats were all three years old with second lactation, and all goats were healthy without diseases and treatment. All procedures were approved by the Institutional Animal Care and Use Committee of College of Veterinary Medicine, Northwest A&F University. Fetal Bovine Serum (10270) and Transferrin (ITS) (1711871) were obtained from Gibco (Waltham, MA, USA). Matrigel (356234) was obtained from BD (Franklin Lakes, NJ, USA). PRL (HY-P71059), osteoprotegerin (OPG) (HY-P71017), RANKL/TNFSF11 (HY-P7425) and Hydrocortisone (HY-N0583) were obtained from MedChemExpress (New Jersey, NJ, USA). Cyclin D1 Monoclonal Antibody (60186-1-Ig), Beta Actin Monoclonal Antibody (66009-1-Ig), PR Polyclonal Antibody (25871-1-AP), ETS-related transcription factor 5 (Elf5) (11155-1-AP,) were purchased from Proteintech (Wuhan, China). Phospho-Stat5 (Tyr694) (C71E5) Rabbit mAb (9314s) was purchased from Cell Signal Technology (Boston, MA, USA).

### 4.2. Paraffin Embedment, Hematoxylin-Eosin (HE) and IHC Staining of Goat Mammary Gland 

Mammary gland samples were fixed in 4% paraformaldehyde (PFA) for 24–48 h. After washed with running water for 12 h, samples were dehydrated with gradient alcohol (50% ethanol for 2 h, 70% ethanol for 2 h, 80% ethanol for 1 h, 95% ethanol for 30 min, absolute ethanol I for 30 min, absolute ethanol II for 30 min), xylene transparent (benzene alcohol for 15 min, xylene I for 3 min, xylene II for 3–4 min), and immersed in wax (immersed in the mixture of 1/2 volume of xylene and soft wax for 30 min, soft wax I for 30 min, soft wax II for 1 h and hard wax I for 40 min, respectively). After tissue embedment, samples were trimmed and sliced (5 μm). HE staining was performed according to the instruction of Solarbio (G1120) (Beijing, China). IHC staining were performed according to the instructions of Solarbio SP (mouse/rabbit IgG)-POD Kit (SP0041). Sections were observed and shot under microscope.

### 4.3. Transmission Electron Microscope Sample Preparation and Dyeing 

After rapid pre-fixation in cold fixation solution (2% PFA + 2.5% glutaraldehyde), tissues were cut into 0.5–1 mm^3^ and formal fixation was performed. After washing three times with phosphate buffered saline (PBS) for 15 min each time, the samples were transferred into osmic acid (diluted with PBS 1:1) for 2–4 h. After that, gradient dehydration was performed on the samples using 30% alcohol, 50% alcohol, 70% alcohol, 80% alcohol, 90% alcohol and 100% alcohol, for 8 min each time. Then, the samples were transferred into osmosis and infiltration solution (alcohol: LR white resin = 3:1 for 2 h, alcohol: LR white resin = 1:1 for 8 h, alcohol: LR white resin = 1:3 for 12 h, LR white resin for 24 h) followed by embedment and polymerization (55 °C 48 h). Uranium dye was added for 20 min (kept away from light) and lead dye was added subsequently for 10 min (avoiding gas) when the copper mesh was clamped in the silica-gel dyeing plate. 

### 4.4. RNA Extraction SOP for Mammary Gland 

The sample of about 60 mg tissue was ground into powder with liquid nitrogen and transferred into a 2 mL tube with 1.5 mL TRIZOL reagent. After homogenized for 2 min, the sample was placed at rest horizontally for 5 min to permit the complete dissociation of nucleoprotein complexes. Then, the sample was centrifuged at 12,000× *g* for 5 min at 4 °C and the supernatant was transferred to a new 2.0 mL tube with 0.3 mL of chloroform/isoamyl alcohol (24:1) addition. The tubes were shaken vigorously for 15 s and centrifuged at 12,000× *g* for 10 min at 4 °C. After centrifugation, RNA remained in the upper aqueous phase. The aqueous phase was transferred to a new 1.5 mL tube with an equal volume of isopropyl alcohol supernatant, mixed well, and placed at −20 °C for 2 h for precipitation. Then, the tubes were centrifuged at 13,600 rpm for 20 min at 4 °C followed by removing the supernatant. The RNA pellet was washed with 1 mL 75% ethanol and re-suspended followed by centrifugation at 13,600 rpm for 3 min at 4 °C. After repeating this, the ethanol was removed completely without disturbing the pellet, and the RNA pellet was air-dried in the biosafety cabinet. To dissolve the RNA pellet, 25 µL~100 µL of DEPC-treated water was added. 

### 4.5. RNA-Seq Library Preparation Protocol (DNBSEQ)

mRNA molecules were purified from total RNA using oligo (dT)-attached magnetic beads and fragmented into small pieces using a fragmentation reagent after reaction over a certain period at the proper temperature. First-strand cDNA was generated using random hexamer-primed reverse transcription, followed by a second-strand cDNA synthesis. The synthesized cDNA was subjected to end-repair and then was 3′ adenylated. Adapters were ligated to the ends of these 3′ adenylated cDNA fragments. PCR products were purified with Ampure XP Beads (AGENCOURT) and dissolved in EB solution. The library was validated on the Agilent Technologies 2100 bioanalyzer. The double-stranded PCR products were heat-denatured and circularized by the splint oligo sequence. The single-strand circle DNA (ssCir DNA) was formatted as the final library. The library was amplified with phi29 to make DNA nanoball (DNB), which had more than 300 copies of one molecular. The DNBs were load into the patterned nanoarray and single end 50 (pair end 100/150) bases reads were generated in the way of combinatorial probe-anchor synthesis (cPAS).

### 4.6. Determination of Serum E_2_, PROG, PRL, RANKL and OPG Levels in Dairy Goats with Firm Udder

The levels of E_2_, PROG, PRL, RANKL and OPG in goat serum were analyzed by ELISA kit. The jugular vein blood of dairy goats with normal and firm mammary glands were collected with anticoagulant-free blood collection vessels. The blood was naturally coagulated at room temperature for 20 min and centrifuged for about 20 min (2500 rpm). After collecting the supernatant, samples were determined by the double-antibody sandwich method using the ELISA kit according to the manufacturer’s instructions of E_2_, PROG, PRL (Jiangsu Meimian Industrial Co., Ltd., Yancheng, China) and RANKL, OPG (Shanghai Bangyi, Shanghai, China).

### 4.7. Primary Culture and RANKL/OPG Treatment of Goat Mammary Epithelial Cells (GMECs)

Mammary gland tissues obtained by the operation of healthy Guanzhong dairy goats were washed with PBS for 3–5 times, and connective and adipose tissues were peeled off. After soaking in a culture medium and cut to 1 mm^3^, acini were inoculated in a 24-well plate and incubated for 6 h at 37 °C, 5% CO_2_ and saturated humidity. DMEM/F-12 medium was added per well and changed every three days. GMECs were cultured for 48 h after passage followed by different treatment for 24 or 48 h with a lactation induction or not. The lactation induction was DMEM/F-12 medium containing 1 μg/mL prolactin, 1 × ITS, 3 μg/mL hydrocortisone and 1 mg/mL BSA. The concentration of RANKL and OPG was 0.5 μg/mL and 5 μg/mL, respectively.

### 4.8. Three-Dimensional Culture of GMECs

Matrigel stored at −20 °C was placed at 4 °C overnight to melt into liquid completely. The bottom of each hole of 96-hole plate was paved with 35 μL matrix and the plate was shaken horizontally to make it evenly spread all over the bottom of holes. Then, the plate was placed in the incubator of 37 °C for 30 min to solidify it. GMECs of 3 × 10^3^ cells per hole were inoculated on the cell-culture plate and cultured under the conditions of 37 °C, 5% CO_2_ and saturated humidity.

### 4.9. Isolation and Culture of Adipose Derived Stem Cells (ADSCs) from Goat Mammary Gland

The fat derived from breast was cut repeatedly using ophthalmic scissors and broken into mealy shapes after washing five times in PBS. Then, the fat was digested with 0.1% type I collagenase at 37 °C for 40 min, and shaken every 10 min. After digestion, the mixture was centrifuged at 2000 rpm for 5 min. The upper semi-solid lipid and middle clear liquid were discarded, and the precipitate was resuspended by culture medium. Then, the last step was repeated and cultured at 37 °C with 5% CO_2_ and saturated humidity.

### 4.10. Induction of Adipogenic Differentiation of ADSCs from Goat Mammary Gland 

When the growth confluence of ADSCs was up to 90%, adipogenic differentiation was induced by the adipogenic induction medium (90% DMEM/F-12 medium + 10% serum + 10 ng/mL IGF + 1 μM dexamethasone + 0.5 mM IBMX + 0.2 mm indomethacin) for 14 d with changed every three days. After induction and being washed twice in PBS wash, cells were fixed with 4% PFA at room temperature for 30 min. Then, the cells were rinsed with 60% isopropyl alcohol for 10 s and incubated with 0.3% oil red O dye solution for 1 min. After discarding the dye solution, cells were rinsed with 60% isopropyl alcohol and PBS, and observed under an inverted microscope.

### 4.11. Induction of Osteogenesis Differentiation of ADSCs from Goat Mammary Gland

When the confluence of ADSCs reached 80–90%, osteogenic differentiation was induced by osteogenic induction medium (90% DMEM/F-12 medium +10% serum +20 nM dexamethasone + 10 mM β-sodium glycerophosphate + 50 μM ascorbic acid) for 14 d with changed every three days. ALP staining: 50 μL chromogenic substrate of alkaline phosphatase kit was added to dish with mixture and incubation at 37 °C for 30 min. The ALP staining was observed under an inverted microscope.

### 4.12. Induction of Chondrogenesis Differentiation of ADSCs from Goat Mammary Gland

When the confluence of ADSCs reached 80% to 90%, cells were digested with 0.1% Trypsin EDTA digestive solution for 2 min. After centrifugation, resuspension and adherence to the wall, chondrogenic differentiation was induced in the ADSCs by chondrogenic induction medium (90% DMEM/F-12 medium + 10% serum + 0.1 μM dexamethasone + 10 mM β-sodium glycerophosphate + 50 μM ascorbic acid + 10 ng/mL TGF-β + 1% ITS) for 14 d with the medium changed every three days. After the chondrogenic induction, cells were washed three times with PBS and fixed with 4% PFA for 30 min. After soaking in 3% glacial acetic acid solution for 5 min, Alcian blue dye solution was added for incubation overnight and then observed under an inverted microscope after being washed with PBS.

### 4.13. Co-Culture of GMECs and Adipocyte-like Cells Induced by ADSCs 

In the co-culture group, Transwell^TM^ chamber (aperture 0.4 μm) was used to establish a co- culture system. After 14 d for 1 × 10^5^ ADSCs of adipogenic induction in the low chamber, GMECs were inoculated 1 × 10^5^ per well in the up chamber, with DMEM/F-12 (added with 10% FBS) added. Cells were incubated at 37 °C, 5% CO_2_ and saturated humidity and the culture medium was changed every three days.

### 4.14. Total Protein Extraction and Western Blotting 

After pre-cooled PBS cleaning twice, a lysis buffer with protease inhibitor was added to the cells after removing the supernatant. Then, the samples were placed on ice for 15 min, shaken quickly, followed by centrifugation of 4 °C and 12,000 rpm for 5 min. The supernatant was the total protein. Before use, samples were mixed with 5× SDS-PAGE sample loading buffer and boiled in boiling water bath for 10 min. Separation gel and concentrated gel were prepared according to the rapid-gel preparation kit instruction and protein samples were loaded. After electrophoresis, the protein gel was transferred to the PVDF membrane (Millipore, Bedford, MA, USA) followed by blocking in 3% BSA of Tris buffer solution for 2 h, then incubated in a primary antibody (1:1000 dilution) at 4 °C overnight and a second antibody (1:3000 dilution) at room temperature for 2 h, and washed three times. The bio rad chemiluminescence kit (Hercules, CA, USA) was used for protein detection. The gray analysis of bands was analyzed by Image J 1.8.0 software (NIH, Bethesda, MD, USA). β-actin was as an internal parameter.

### 4.15. RNA Isolation and Quantitative Real-Time PCR (qRT-PCR) Analysis

In each group, cells were pooled for RNA extraction. Reverse-Transcription PCR (RT-PCR) was carried out using Evo M-MLV Mix Kit with gDNA Clean for qPCR (AG, Changsha, China) according to manufacturer’s instructions. qRT-PCR was performed using Real-time Detection System (QuantStudio 6 Flex) (ABI, Singapore) and SYBR^®^ Green Premix Pro Taq HS qPCR Kit (ROX Plus) (AG, Changsha, China). Each experiment was repeated independently at least three times, and the fold change in the expression of each gene was analyzed using the 2^−ΔΔCT^ method. All primers were used as the Supplementary Table S1 [60,61]. β-actin served as a reference gene. 

### 4.16. Immunofluorescence Staining

GMECs were fixed in 4% PFA at room temperature for 30 min. After being washed with PBS 3 times, cells were permeated with 0.2% Triton X-100 (dissolved in PBS) 10 min. After being washed with PBS 3 times, cells were blocked in 1% FBS solution (dissolved in PBS) at room temperature for 1 h. Then cells were incubated in primary antibodies of p-stat5 and Elf5 (1:100 dilution) at 4 °C overnight and secondary antibody (1:100 dilution) at room temperature for 2 h away from light. After being washed with PBS 3 times, cells were transferred to Hoechst (1:1000 dilution) for 5 min and placed under the fluorescence microscope for a micrograph.

### 4.17. Statistical Analysis

The experimental data were analyzed by the Student *t* test (the means of two groups) or one-way ANOVA using SPSS 19.0 statistical software (IBM, Amenk, NY, USA). Mean (Mean) + standard error (S.E) was used. *p* < 0.05 was considered significant.

## 5. Conclusions

There is a lower milk yield in dairy goats with firm udders, and this study was conducted to explore the abnormal phenomenon and underlying mechanism contributing to firm udders in order to provide a foundation for preventing its formation and improving milk production. This study revealed the structure of firm udders with developed connective tissue and smaller acini per lobule during lactation and confirmed that there were lower serum levels of E_2_ and PROG, and higher mammary expression of ERα and PR, in dairy goats with firm udders. Further research suggested that the upregulated PR/RANKL signaling pathway contributed to the occurrence of, and decreased milk production in, firm udders. Co-culturing with adipocyte-like cells could increase the RANKL expression and acini formation of GMECs, and the high RANKL level of dairy goats with firm udders could promote GMECs proliferation and decrease lactoprotein synthesis with a slightly negative impact on acini formation. Thus, decreasing the RANKL level of dairy goats to prevent firm udders and improve udder health and milk yield is worth further investigation.

## Data Availability

All data generated or analyzed during this study are included in this article.

## Supplementary Materials

The following supporting information can be downloaded at: https://www.mdpi.com/article/10.3390/ijms24108841/s1.
