# Supplementary material for: The High Level of RANKL Improves IκB/p65/Cyclin D1 Expression and Decreases p-Stat5 Expression in Firm Udder of Dairy Goats
Source: Int J Mol Sci. 2023 May 16;24(10):8841. doi: 10.3390/ijms24108841 (PMC10218954; doi:10.3390/ijms24108841)
Supplement: Supplementary file 1 [file ijms-24-08841-s001.zip › ijms-2359000-supplementary.pdf]

Table S1. Primer sequence

Genes	Primer sequences (5'-3')	Source
ERα	CGGTGGATGTGGTCCTTCTCT<br>AGGGAAGCTCCTATTTGCTCC	This manuscript
ERβ	GCTAACCTGCTGATGCTCCTGTCTC<br>GCCCTCTTTGCTCTCACTGTCCTC	This manuscript
PR	GAGAGCTCATCAAGGCAATTGG<br>CACCATCCCTGCCAATATCTTG	This manuscript
PRLR	GCTGGAGAATCCCGAAAC<br>AGCAGAGTGGCTGTGGTG	This manuscript
HSP70	ACGTTCGACGTGTCCATTCT<br>TCACCAGCCTGTTGTCGAAG	This manuscript
HSP90	CAAGAGCCTGACCAACGACT<br>AAAGGAGCTCGTCTTGGGAC	This manuscript
CREB	CACTCAGCCAGGCACTACCA<br>GGAAGACGCCATAACAACCC	[60]
RANK	GGGTTGCCATGAACTATCAGTGA<br>AACGATCAAAGCAACCAGTTTTTA	[61]
RANKL	TTCAGAATTCCCCAGCCAGTA<br>CCAAAACCAGCATCAAAATCC	[61]
Cyclin D1	CCTGCCGTCCATGCGGAA<br>GAACTTCACATCTGTGGCAC	This manuscript
β-actin	GTCACCAACTGGGACGACAT<br>CATCTTCTCACGGTTGGCCT	This manuscript
